# Integration of palaeo-and-modern food webs reveal slow changes in a river floodplain wetland ecosystem

**DOI:** 10.1038/s41598-020-69829-8

**Published:** 2020-07-31

**Authors:** Giri R. Kattel, Bradley D. Eyre, Peter A. Gell

**Affiliations:** 1 School of Sciences, Psychology and Sport, Federation University Australia, Mt. Helen, VIC 3350 Australia; 2 Water, Environment and Agriculture Program, Department of Infrastructure Engineering, The University of Melbourne, Victoria, 3010 Australia; 3 Resilience and Transformation Centre in China, Nanjing Institute of Geography and Limnology Chinese Academy of Sciences, 73 East-Beijing Road, Nanjing, 210008 China; 4 Department of Hydraulic Engineering, Tsinghua University, Beijing, 100084 China; 5 Centre for Coastal Biogeochemistry, School of Environmental Science and Engineering, Southern Cross University, Lismore, NSW 2480 Australia

**Keywords:** Stable isotope analysis, Ecosystem ecology

## Abstract

Large rivers, including the Murray River system in southeast Australia, are disturbed by many activities. The arrival of European settlers to Australia by the mid-1800s transformed many floodplain wetlands of the lower Murray River system. River impoundment and flow regulation in the late 1800s and, from the 1930s, resulted in species invasion, and elevated nutrient concentrations causing widespread eutrophication. An integrated palaeoecology, and palaeo-and-modern food web approach, incorporating mixing models, was undertaken to reveal changes in a regulated wetland (i.e. Kings Billabong). The lack of preserved sediment suggests the wetland was naturally intermittent before 1890. After this time, when used as a water retention basin, the wetland experienced net sediment accumulation. Subfossil cladocerans, and δ^13^C of *Daphnia*, chironomid, and bulk sediment, all reflected an early productive, likely clear water state and shifts in trophic state following river regulation in the 1930s. Food web mixing models, based on δ^13^C and δ^15^N in subfossil and modern *Daphnia*, fish, and submerged and emergent macrophytes, also indicated a shift in the trophic relationships between fish and *Daphnia*. By the 1970s, a new state was established but a further significant alteration of nitrogen and carbon sources, and trophic interactions, continued through to the early 2000s. A possible switch from *Daphnia* as a prey of Australian Smelt could have modified the food web of the wetland by c. 2006. The timing of this change corresponded to the expansion of emergent macrophytes possibly due to landscape level disruptions. The evidence of these changes suggests a need for a broader understanding of the evolution of wetlands for the management of floodplains in the region.

## Introduction

Large river floodplain wetland systems have complex food webs that support significant ecosystem goods and services^1^. However, these large river floodplain wetlands are impacted by extreme climate change, and anthropogenic disturbances such as increased river regulation and water pollution^2^. The integration of palaeoecology, modern ecology and carbon energy and nutrient mass flow approaches to understand food web dynamics can resolve many key questions of the modern ecosystem structure and function of shallow floodplain wetland systems^3,4^. This combined approach provides an historic perspective that should facilitate best practices for the management of the hydrology and ecology of regulated river systems^5^.

The lower Murray River system in southeast Australia has witnessed rapid hydrological transformation over the past century. This has impacted biodiversity and ecosystem structure and the function of wetlands across temporal and spatial scales^6^. The majority of these wetlands have shown marked declines in submerged aquatic macrophyte communities following the arrival of Europeans in the mid-1800s^7^. River regulation and widespread catchment modifications for agriculture are implicated in driving these changes^8^ but waste from mining and settlements^9–11^ has also had considerable impact on most river tributaries. The variability in river flows, from both natural and anthropogenic, together with increased nutrient and sediment fluxes and a subsequent decline in water clarity significantly reduces underwater light and photosynthesis^7,8,12^.

The cascading effects of eutrophication and disruption in the connectivity with the river system have driven unprecedented changes in floodplain wetland ecosystems^13^. The changes include decline in the water level of some wetlands, and perennial inundation of others followed by the partial or complete loss of sub-merged vegetation as well as the proliferation of fringing emergent macrophytes (*Typha orientalis*, *T. domingensis* and *Phragmites australis*)^14^ and floating plants (e.g. *Azolla* sp., *Lemna* sp.) and the invasion of exotic carp (*Cyprinus carpio*)^15^. Alteration of carbon and nitrogen sources in the system have modified the food web structure and trophic pathways^16^. Particularly, the increased disturbance of terrestrial and pelagic or periphytic habitats has altered carbon energy and nitrogen mass flows from the base of the food web to higher trophic levels^17,18^.

Various monitoring approaches have been employed to better understand the changes in floodplain wetland ecosystems in southeast Australia to inform management practices designed for their restoration. For example, the water requirements of riparian forests has been quantified to mitigate the impact of water resource development and drought on keystone river red gum communities^19^. The macroinvertebrate community composition and densities have been monitored to assess low flow impacts as they are seen as critical in the successful recruitment of native fish and water birds^20^. Similarly, palaeolimnological approaches have been used to assess the changes in water quality due to excessive nutrients such as total phosphorous (TP) and total nitrogen (TN) loads and salinization and acidification (pH) in the region over the past century^21^. However, these approaches have not explored the changing nature of food web dynamics of the Murray River floodplain wetland system over the recent past.

Palaeoecology provides evidence of benchmarks for undisturbed ecosystems, and can show how the system has responded to change following a disturbance, while modern ecology defines current system behaviour, including linkages across trophic pathways^22–25^. The carbon energy and nutrient mass flows in floodplain wetlands provide information on consumer food sources and trophic changes over time^26,27^. The combination of subfossil cladocerans, such as chydorids, bosminids and daphnids, and the stable isotope analysis (SIA) of carbon and nitrogen in primary producers (e.g. aquatic macrophytes) and primary and secondary consumers (zooplankton and fish), is likely to prove a useful tool for understanding the contemporary condition of floodplain wetlands which can be used to underpin a management framework in the lower Murray River system^5,28^. The variability in the conditions can be achieved through the use of mixing models that provide critical information regarding changing food sources and predator–prey interactions^25,26,29,30^. However, the dynamics of stable isotopes of carbon and nitrogen across the trophic levels are complex. This is largely dependent on the type and pool source of the organic matter and the fractionation of elements during the movement from lower to higher trophic levels^31^.

In this study, we have integrated palaeoecology and palaeo-and-modern food web approaches to assess recent changes in Kings Billabong, a shallow regulated floodplain wetland, in the lower Murray River system of southeast Australia, by using carbon and nitrogen stable isotopes. We hypothesize that wetland impoundment due to river regulation by early European immigrants has led to the collapse of sub-merged vegetation leading to a change in the source of carbon. The integration of cladoceran palaeoecology, the SIA of carbon in subfossil *Daphnia* and bulk sediment, as well as SIA of carbon and nitrogen in contemporary submerged and emergent macrophytes, and modern *Daphnia* and fish, provides key evidence for the nature of wetland change including nutrient (carbon and nitrogen) movements across the trophic levels.

## Materials and methods

### Study site

**Figure 1:**
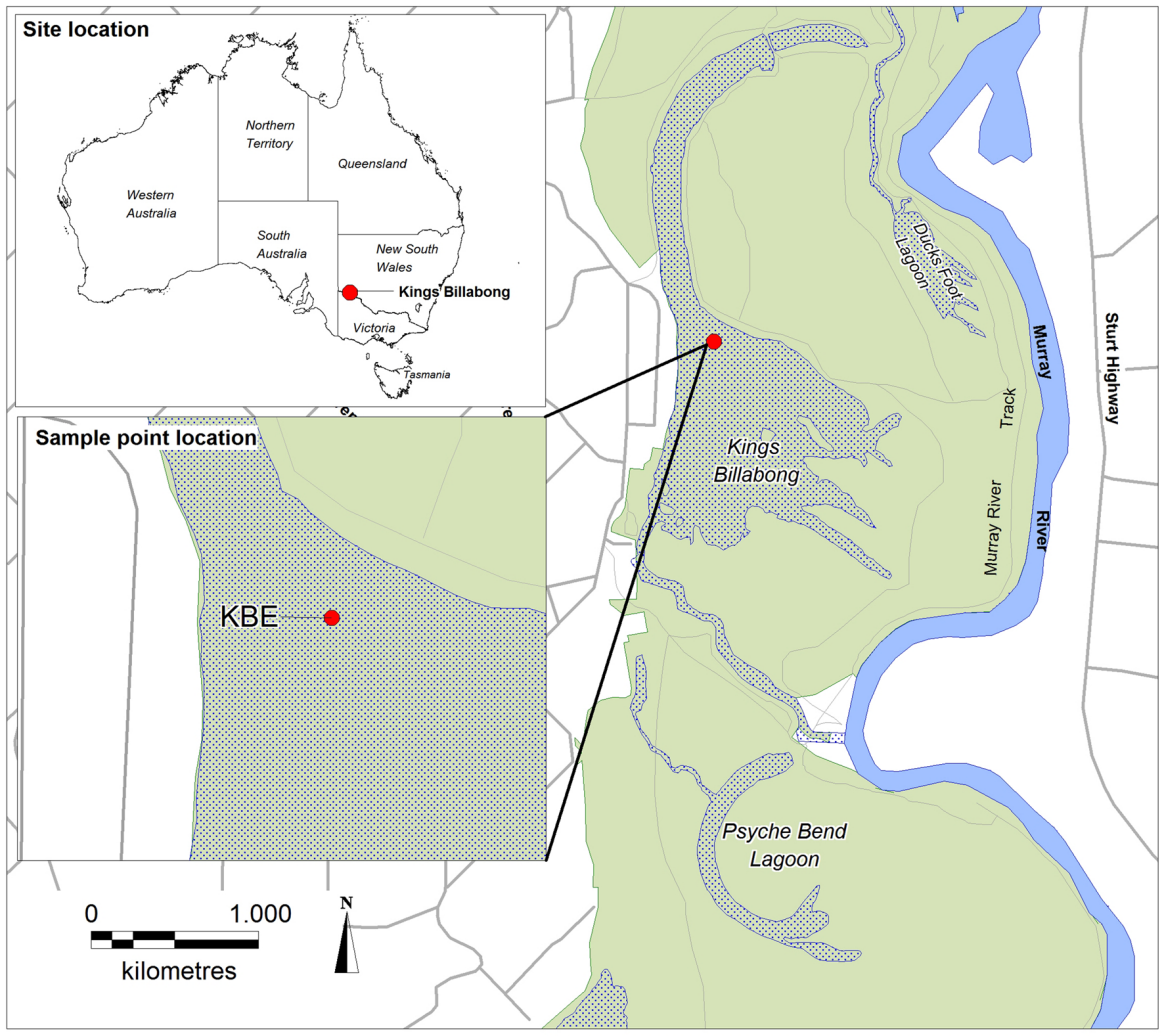
Map of Kings Billabong wetland showing the deepest point (2.3 m) where the sediment core was collected. The shaded areas are the areas of water, green areas are catchment emergent aquatic macrophytes and terrestrial catchment vegetation.

Kings Billabong is a shallow (max. depth = 2 m) wetland of the lower Murray River system in northwest Victoria (34° 11′ 0″ S, 142° 09′ 0″ E) (Fig. 1). The ecosystem of Kings Billabong was likely to have been modified after it was used as a water storage basin from the early 1890s^32,33^. Construction of the pumping station on the River at Psyche Bend, in 1891, enabled the filling of Kings Billabong for the emerging irrigation industry. Prior to the pumping station the connectivity of Kings Billabong with the Murray River was natural, influenced largely by regular flooding and drying patterns. After 1891, the Billabong became permanently inundated for the first time with pumping from the river maintaining water levels during dry times. Flow regimes in the Murray River channel itself were interrupted severely following the phase of river regulation after the construction of locks along the river during the 1920–1930s^34^. The regulation triggered changes in the condition of aquatic ecosystems and the floodplains through both reduced overbank flooding and the permanent filling of wetlands linked to weir pools. For example, the river red gum (*Eucalyptus camaldulensis*) forests around the Billabong began to die through inundation^19^. There was also a gradual decline in the cover of the submerged aquatic plant species such as, *Vallisneria americanus* and charophyte species such as *Nitella sonderi*, *N. hyalina*, *N. stuarti*, and *Chara gymnopytis* (unpublished subfossil records) as well as the development of dense stands of *Typha domingensis* and *Phragmites australis* around the wetland margins^35^. Over the decades after river regulation, the relatively shallow Kings Billabong did not respond abruptly, but underwent a gradual and sustained phase of ecosystem degradation^33^. The slow and sustained response in ecological state to the gradual change of the wetland environment led to the collapse of the submerged vegetation stands during the late twentieth century^33^. The first sign of change was visible with the growth of *Typha* sp. during the 1950s. The aerial photos show the areas of emergent macrophytes began to expand rapidly around the wetland margins during the 1970s.

### Analysis of subfossil cladocerans from the sediment core

A 94 cm long sediment core was retrieved from the centre of Kings Billabong wetland in 2011. A central core was taken to represent conditions across the wetland and the sediments proved to preserve microfossils of both littoral and planktonic cladoceran zooplankton^36^. The core was then subsampled at high resolution (1 cm interval) for subfossil cladoceran analysis. Sub-samples were treated with 10% KOH solution, and heated at 60 °C on a hotplate for at least 45 min. The subsample mixture was then sieved through a 38 µm mesh. Safranin was used to stain the remains before the slides were prepared^42^. Cladoceran remains were counted under 400 × magnification and identified and described following^37,38^. Cladoceran species were classified as ‘littoral’ and ‘planktonic’ according to their preference to water quality and habitats and the grouping was tested using constrained cluster analysis (CONISS)^39^.

### Collection and analysis of subfossil ephippia of cladocerans (*Daphnia*)

Subfossil *Daphnia* ephippia were used for the analysis of δ^13^C and δ^15^N. The ephippia reflect the stable isotopic composition of the parent *Daphnia*, their diet and the environmental water in which they have lived^40^. The δ^13^C of *Daphnia* ephippia in different seasons has also been found to match the δ^13^C of their body parts^41^. Based on the assumption of the close relationship of the δ^13^C between *Daphnia* ephippia and their body parts, altogether six subfossil *Daphnia* sub-samples were analysed. These were taken at the depth intervals of 0–10 cm, 10–20 cm, 20–30 cm, 30–40 cm, 40–50 cm, 50–60 cm, respectively from the same core used for subfossil cladoceran analysis^32^. Prior to picking *Daphnia* ephippia, the sediment was treated with 10% KOH as described in Kattel et al.^42^. *Daphnia* ephippia were identified under a binocular microscope following^43^.

### Collection/preparation of modern aquatic emergent macrophytes, fish and Cladocera (*Daphnia*)

For the SIA of C and N of fish: Murray cod (*Maccullochella peelii*), common carp (*Cyprinus carpio*) and Australian smelt (*Retropinna semoni*) were collected using gill and seine nets in March 2014. Living *Daphnia* were collected from Kings Billabong wetland using hand-nets (100 µm mesh size) from both open and littoral areas on the day the fish were collected.

For vegetation samples, both upper and lower sections of the stem and leaves of the two dominant stands of emergent aquatic macrophytes: *Typha domingensis* and *Phragmites australis*, and one sub-merged charophyte species, *Nitella sonderi*, were collected from the littoral zone at the same time. The upper and lower sections of the plantstand and leaves were selected to ensure that the isotopic compositions would be representative of the whole plant. Each sample was washed and transported to the laboratory.

Murray cod and common carp, once caught by gill nets, were placed live in a submerged cage overnight, while the Australian smelt was collected using seine nets, and transported to the laboratory. Live *Daphnia* were placed in an aquarium for 24 h to void their gut contents. Cladoceran zooplankton were acid washed with 10% HCl and rinsed in distilled water to avoid contamination of the organic carbon from the carbonate fraction^44^. All biological materials were oven dried at 30 °C for 72 h before they were pulverised and submitted for SIA^32^.

### Dating of sediment core

The biological remains, including subfossil cladocerans, were thought to be mixed in the water column before being deposited in the sediment at 2–3 m depth in Kings Billabong, where the 94 cm long 80 mm piston core was collected in 2011. Rather than from the lake centre, the core collected from the deepest point, as the deepest area of the wetland is usually the best for studying palaeoecology due to the integration of both littoral and pelagic cladoceran zooplankton microfossils^36^.

The ^210^Pb-based age model was established from a total of nine subsamples^45^ down to 51 cm in the sediment core based on both constant initial concentration (CIC) and constant rate of supply (CRS) models. The age-depth model for the rest of the core was established using extrapolation methods^46^. This analysis was undertaken at the Australian Nuclear Science and Technology Organization (ANSTO), Lucas Heights, Sydney. More details of dating method is described in a previous study on Kings Billabong^33^.

### Analysis of δ^13^C and δ^15^N of emergent and sub-merged macrophytes, fish, and modern and subfossil cladoceran zooplankton

Each dry sample (both modern macrophytes, *Daphnia*, fish and subfossil *Daphnia*), weighing 100–200 µg, was packed into a small tin cup for isotope analysis. The subfossil *Daphnia* ephippia samples were oxidised and the resultant CO_2_ and N_2_ analysed with a Finnigan Delta Plus mass spectrometer interfaced via a Conflo II to a NC2500 Elemental Analyzer. The δ^13^C, δ^15^N isotopic values, were determined using a Thermo Finnigan Flash EA 112 interfaced via a Thermo Conflo III with a Thermo Delta V Plus IRMS^47,48^. Samples of acetanilide of known isotope composition were analyzed within each run to verify isotope values. Reproducibility for both δ^13^C and δ^15^N was 0.2‰ while that for both %C and %N was 1%^47,48^. Ratios of ^13^C/^12^C and ^15^N/^14^N were expressed as the relative per mil (‰) difference between the sample and conventional standards (PDB carbonate and air N_2_, respectively). These ratios were expressed as: δX = [R (sample)/R (standard) − 1] × 1,000 (‰), where X = ^13^C or ^l5^N and R = ^13^C/^l2^C or ^l5^N/^14^N^32^.

The modern *Daphnia* isotopic values of nitrogen were derived from the samples collected in 2014, while the subfossil *Daphnia* isotopic values of nitrogen were from the top sections (0–10 cm) of the core that dated back to c. 2006–2011.

### Analysis of δ^13^C and δ^15^N values of bulk sediment

Analysis of δ^13^C and δ^15^N values was also carried out to compare with the stable isotope values of C and N of subfossil *Daphnia* and chironomids. The method for the analysis of δ^13^C values for 45 bulk sediment samples of the core can be found in detail in Kattel et al.^32^. Details of the analysis of δ^15^N for the same number of bulk sediment samples can be found in Kattel et al.^33^. Samples, once weighed (c. 100–200 µg), were treated with 1 mL HCl to remove carbonates and then analyzed on an Elementar VarioMICRO Elemental Analyser and a Continuous Flow Isotope Ratio Mass Spectrometer (GV Instruments IsoPrime). The acid-treated fraction and results of δ^13^C were normalized to the reference standard IAEA C8 (consensus value: δ^13^C_V-PDB_ = − 18.31‰)^49^. All δ^15^N analyses were performed on the untreated fraction and a two point normalization was performed on the data using international reference standards IAEA N-2 (consensus value: δ^15^N_AIR_ =  + 20.3‰ and USGS-25 (consensus value: δ^15^N_AIR_ = − 30.4‰)^50^.

### Food web mixing model

An integrated palaeoecological, and palaeo-and-modern food web analysis approach was used to test the changes of wetland trophy, and the trophic pathways of the food web in Kings Billabong. The subfossil assemblages of cladocerans were used to infer trophic changes of the wetland before and after river regulation. However, key questions remained and these are the focus of this study, namely how the carbon and nitrogen sources of cladocerans in the past compare with those at present, , and how the palaeo- and modern cladoceran diets differ with respect to basal food sources and how are the isotopic signatures of carbon and nitrogen transferred to upper trophic levels of the ecosystems. Hence, the contemporary and past responses of wetland ecosystem state and food web dynamics were evaluated by using a mixing model of stable isotopes of carbon and nitrogen from both modern macrophytes, cladoceran zooplankton (*Daphnia*), fish (large vs small) and subfossil *Daphnia*^51^. The linkages among cladocerans and other biota, including fish and macrophytes, were then established by applying stable isotope values of carbon and nitrogen of each organism to a mixing model.

Mixing models are mathematical equations (Eqs. 1 and 2), often applied in analyses of carbon and nitrogen stable isotopes. The models describe the observed isotopic composition as a mixture of the assimilated isotopic compositions based on the isotopic mass balance^29^. Individual isotopic values of δ^13^C and δ^15^N are measured in primary producers and consumers using mixing models. For example, the mixing (combined) values of the carbon isotopes are given in the equations below:

1 $$\begin{aligned} & \delta 13Cmix = f1\delta 13C1 + f2\delta 13C2, \\ & f1 + f2 = 1 \\ \end{aligned}$$

where, δ^13^C_mix_ is the isotope signature of the consumer, usually a combination of the δ^13^C of individuals (e.g. subscripts of Carbon i.e. 1 and 2 are combination of two individuals usually prey 1 and prey 2), weighted by their unknown diet fractions (*f*_*1*_ and *f*_*2*_, respectively). Assuming that the combination of diet fractions is 1, then *f*_*1*_ and *f*_*2*_ are usually estimated algebraically by rearranging the equations to:

2 $$\begin{aligned} & f1 = \frac{{\left( {\delta 13Cmix - \delta 13C2} \right)}}{{\left( {\delta 13C1 - \delta 13C2} \right)}}, \\ & f2 = 1 - f1 \\ \end{aligned}$$

The isotopic fractionation across trophic levels is much smaller for carbon isotopes (range = 0–l‰ per trophic level)^52^. The measurement of mean δ^13^C values primarily indicate the basal source of carbon generated by primary producers in the food web that is important for the energy of higher-level consumers^53,54^. Hence, the δ^13^C values are used for the information about consumer food preferences. Unlike carbon, the isotopic fractionation for nitrogen is much larger (range = 1.3–5‰ per trophic level), hence the measurement of mean δ^15^N is used as evidence for the number of trophic levels present in a food web^55^.

## Results

### Age modelling

**Figure 2:**
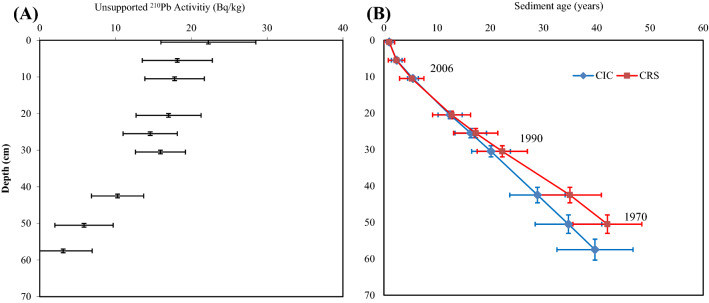
(**a**) King Billabong core unsupported ^210^Pb activities plotted against depth, (**b**) King Billabong core CIC and CRS model ages plotted against depth.

There was a very small variation between the supported ^210^Pb (sediment containing a background level of ^210^Pb by the decay of ^226^Ra eroded from rocks) and unsupported ^210^Pb (sediment already with excess of ^210^Pb, occurring through the decay of ^238^U followed by ^222^Rn in the atmosphere then to ^210^Pb, washed out of the atmosphere) (Table 1). The constant rate of sediment accumulation model (CRS) suggested that, at 51 cm sediment depth, the rate of accumulation was 0.6 g cm^−2^ year^−1^ yielding a corresponding age of c. 1970 (Fig. 2a,b). A linear extrapolation approach^46^ of SAR showed that the sediments at 75 cm were deposited in c. 1930.

**Table 1:** The ^210^Pb dating of the sediment core collected from Kings Billabong.

Depth (cm)	Cumulative dry mass (g/cm^2^)	Total 210Pb (Bq/kg)	Supported ^210^Pb (Bq/kg)	Unsupported ^210^Pb (Bq/kg)	Unsupported ^210^Pb (Bq/kg)	Calculated CIC ages (years)	Calculated CRS ages (years)	CRS mass accumulation rates (g/cm^2^/year)
0–1	0.5 ± 0.5	60 ± 4.8	38.3 ± 3.7	21.7 ± 6.1	22.2 ± 6.3	0.4 ± 0.4	0.4 ± 0.1	1.2 ± 0.3
5–6	5.5 ± 0.5	56 ± 2.5	38.3 ± 3.7	17.7 ± 4.5	18.1 ± 4.6	4 ± 1	5 ± 3	1.2 ± 0.3
10–11	10.5 ± 0.5	48.9 ± 2.6	31.6 ± 2.8	17.3 ± 3.8	17.8 ± 3.9	8 ± 2	9 ± 3	1.1 ± 0.2
15–16	15.5 ± 0.5	50.9 ± 2.6	28.8 ± 2.7	22.1 ± 3.8	22.7 ± 3.9	11 ± 3	14 ± 3	0.7 ± 0.1
20–21	20.5 ± 0.5	46.5 ± 2.2	37 ± 3.6	9.6 ± 4.2	9.8 ± 4.3	15 ± 4	19 ± 3	1.4 ± 0.6
25–26	25.5 ± 0.5	44.6 ± 2.1	30.4 ± 2.8	14.2 ± 3.5	14.6 ± 3.6	18 ± 5	24 ± 3	0.8 ± 0.2
30–31	30.5 ± 0.5	48.3 ± 2.1	33 ± 2.4	15.3 ± 3.2	15.9 ± 3.3	22 ± 5	31 ± 3	0.6 ± 0.1
42–43	42.5 ± 0.5	44.9 ± 2.1	35 ± 2,6	9.9 ± 3.3	10.3 ± 3.4	30 ± 7	52 ± 4	0.5 ± 0.1
50–51	50.5 ± 0.5	45. ± 2.1	40.1 ± 3.1	5.6 ± 3.7	5.8 ± 3.8	36 ± 9	67 ± 4	0.6 ± 0.1

### Palaeoecology of cladocerans

**Figure 3:**
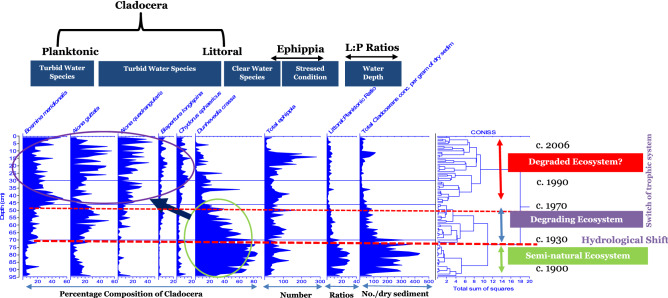
Trophic changes of Kings Billabong revealed by the percentage abundance of the most common planktonic and littoral cladocerans. The assemblage of planktonic species prior to river regulation (1930) is low. River regulation caused hydrological shift of the Murray River causing (low littoral:planktonic ratios) as well as the composition of *B. meridionalis* has increased. Among littoral cladocerans, the assemblage of turbid water preferring species mostly small *Alona*, *A. guttata*, and other species, *A. quadrangularis*, *Biapertura longispina* and *C. sphaericus* all have increased, but the composition of the pioneer clear water macrophyte preferring species, *D. crassa*, declined from c. 1970. The post 1990 shows a sign of reduced water quality due to increased turbidity revealed by increased cladoceran ephippia. Depth information in the y-axis (in cm) is from the sediment core.

Change in the ecosystem of Kings Billabong wetland was revealed by shifts in the composition of assemblages of subfossil cladocerans over time (Fig. 3). As the wetland was used as a water storage basin from c. 1891 the record of change commenced from this time. This early phase of inundation supported a diverse cladoceran fauna that reflects a healthy community. Subfossil cladoceran abundances suggest relatively clear water existed in Kings Billabong wetland between c. 1891 and c. 1930 (Fig. 3). There appears to have been limited influence of the pumping of water into Kings Billabong on the wetland condition during this time.

The principal evidence of clear water during this period is the high relative abundance of the littoral cladoceran, *Dunhevedia crassa,* a species preferring clear water conditions. Despite this prevailing state, relative to *D. crassa*, species tolerant of turbid water condition, such as the planktonic species *Bosmina meridionalis*, and the littoral forms *Alona quadrangularis* and *Biapertura longispina,* showed an upward trend of relative abundance for a short period indicating the response to the occasional flooding of the Billabong and subsequent inundation leading to a nutrient-rich environment, albeit temporarily (Fig. 3).

The most obvious change detected in the subfossil cladoceran assemblages coincides with river regulation from c. 1930. The aquatic ecosystem of Kings Billabong from this time began to change steadily. This phase of degradation was sustained until 1970. While this wetland was artificially inundated from 1891, the onset of this change in the ecology of the wetland occurred particularly after the modification of the natural hydrology of the Murray River itself from ~ 1930, when the effects of river regulation were established. From this time, the abundance of *D. crassa* began to decline, while the abundance of small *Alona*, such as *Alona guttata* and the planktonic *B. meridionalis,* increased. This trend was also matched by sustained declines in the littoral to planktonic (L:P) ratio and inferred reduction in water clarity (Fig. 3).

By c. 1970, the abundance of the littoral, plant-preferring species *D. crassa* markedly declined reflecting a significant loss, or possibly a ‘collapse’, of sub-merged aquatic vegetation in Kings Billabong. A rapid growth and development of an emergent and fringing macrophyte community, such as *Typha* sp. and *Phragmites* sp., as well as increased invasion of common carp, was recorded at or around this time^56,57^. The increasing ecological stress, such as the disappearance of submerged macrophytes, which began in 1930, followed by prolonged eutrophication was reflected by the highest concentrations of subfossil cladoceran ephippia in the sediment from this time until the late 1990s (Fig. 3).

### The δ^13^C values in subfossil *Daphnia*, subfossil chironomids and bulk sediments

**Figure 4:**
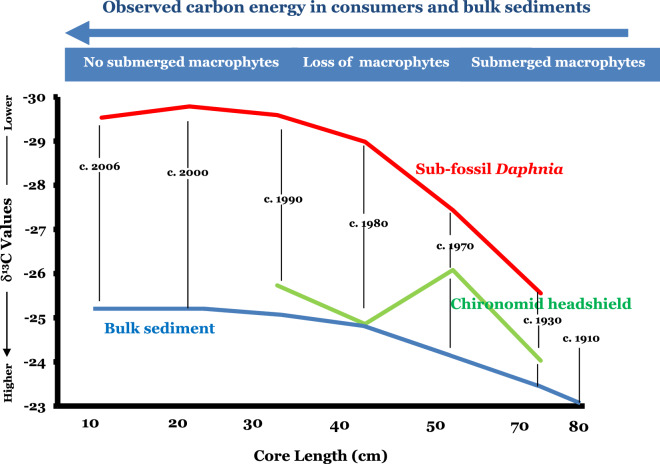
The δ^13^C values extracted from subfossil *Daphnia* ephippia, chironomid headshields and bulk sediment samples from Kings Billabong. A marked shift in carbon source is revealed over time in all three proxies. From 1910 to 1930, the source of carbon was relatively high (δ^13^C > − 23‰). From 1930 to 1970 and 1980 the carbon source continued to decline, and was variable among the proxies; and after 1980 the carbon source further depleted in all proxies. The depleted δ^13^C values also corresponded to the change in the dynamics of subfossil littoral cladoceran (e.g. *D. crassa*) assemblage as shown in Fig. 3 reflecting the possible reduction in the sub-merged macrophyte-derived carbon source for primary and secondary consumers.

The δ^13^C values of subfossil *Daphnia*, subfossil chironomids and bulk sediment in the Kings Billabong wetland sediment core began to decline after c. 1910s (Fig. 4). All three proxies showed a relative depletion of δ^13^C values along with the gradual loss of the sub-merged macrophyte community over time (1910–2006). The δ^13^C values in bulk sediments were the highest followed by those from subfossil chironomids and subfossil *Daphnia*. After river regulation, the δ^13^C values in both consumers depleted reaching as low as − 29.5‰ in the 2000s. However, unlike the subfossil *Daphnia*, the subfossil chironomid δ^13^C values showed a relatively close relationship with the bulk sediment δ^13^C values (Fig. 4).

### Food web mixing models

**Figure 5:**
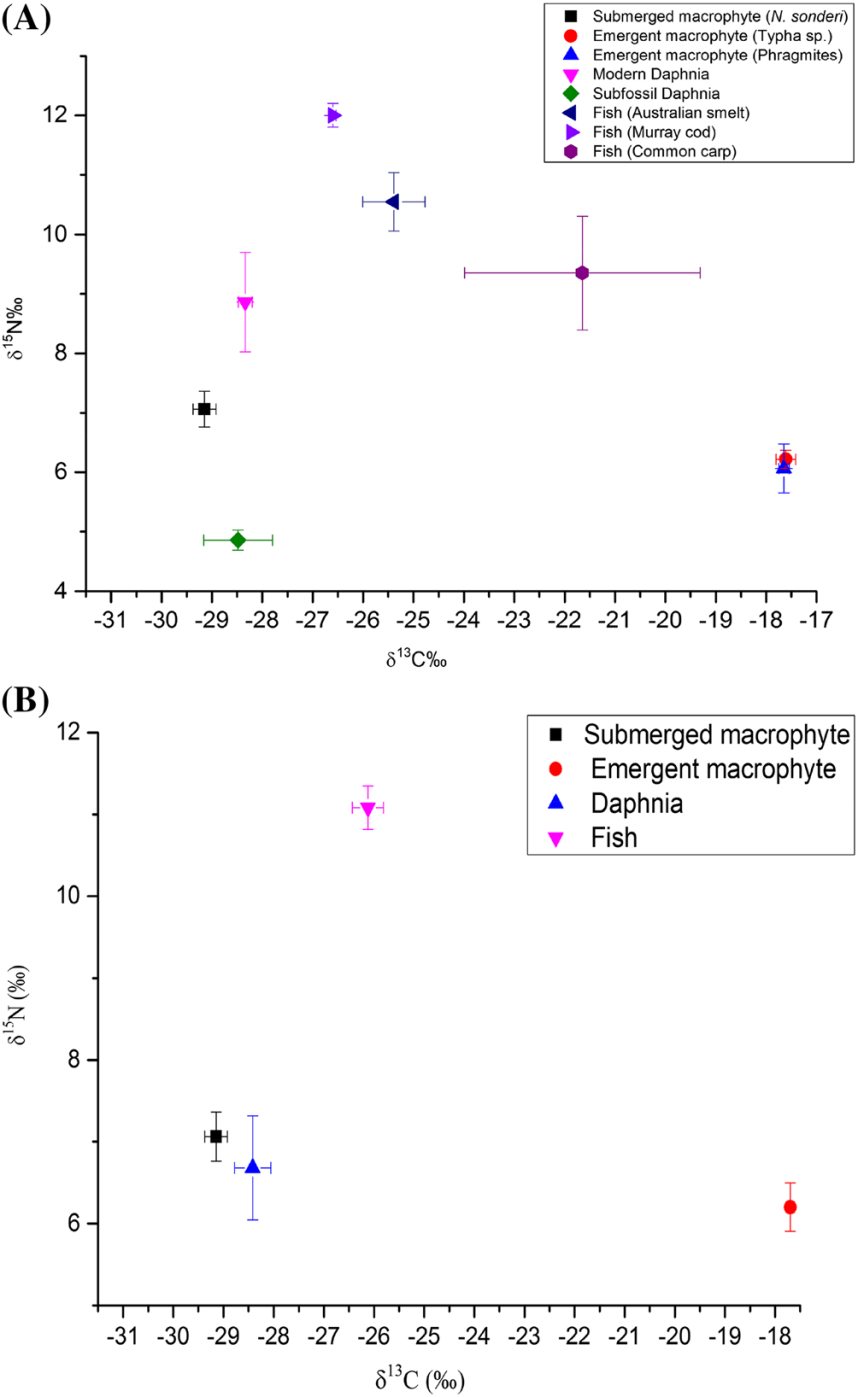
Isotopic representations of the mean (± SE) of δ^13^C and δ^15^N in Kings Billabong derived in mixed models. (**A**) comparison of individual species of fish (Australian smelt, Murray cod and common carp), *Daphnia* (modern and subfossil), sub-merged macrophyte (*N. sonderi*) and emergent macrophytes (*Typha* and *Phragmites*); (**B**) comparison after all fish species, all *Daphnia* and all emergent macrophytes are combined.

Food web mixing models of fish, modern and subfossil *Daphnia* and submerged and emergent aquatic macrophytes revealed different relationships among consumers and primary producers in Kings Billabong (Fig. 5a,b). According to the isotopic fractionation of carbon (~ 1‰) and nitrogen (~ 3.4‰) at each trophic level^58^, the δ^13^C values between modern *Daphnia* and a submerged macrophyte (*N. sonderi*), and the δ^15^N values between modern *Daphnia* and Murray cod, showed a relatively close trophic relationship (Fig. 5a). The difference in the mean δ^15^N values between modern (8.9‰) and subfossil (4.6‰) *Daphnia* was apparent as obviously the modern *Daphnia* utilized more enriched nitrogen sources in Kings Billabong over recent time than the subfossil *Daphnia* 5 years prior.

The mean δ^13^C values of all fish species, *Daphnia*, emergent and submerged macrophytes showed that the transfer of carbon from the base of the food web to *Daphnia* may have occurred mainly from the submerged macrophytes community, while the mean δ^15^N values in most biota revealed that submerged and emergent macrophytes and *Daphnia*, all may have contributed nitrogen masses to the fish community in Kings Billabong (Fig. 5b).

## Discussion

### Changes in the ecology of Kings Billabong

The integrated approach of analysing modern and subfossil biotic assemblages and stable isotopes of carbon and nitrogen in different biota and bulk sediment reveals a sustained change in the ecology of Kings Billabong after the artificial inundation of the wetland in 1891. The timing of the onset of sediment accumulation coincides with the pumping of water from the nearby Psyche Bend into the Billabong for irrigation purposes. The onset of pumping converted the wetland from a naturally intermittent to a permanently inundated basin allowing for the preservation of a continuous sediment sequence. Most intermittent sites in the region today are dominated by semi-terrestrial plants and aquatic communities that grow rapidly once inundated. The water quality effect on those sites is usually localized and dependent on the degree of disturbance, both anthropogenic and climatic.

#### 1891–1930 AD

The onset of permanent inundation, together with rainfall events during the mid-1890s, was a significant change enabling the protection of soft sediments in Kings Billabong^59,60^. A relatively stable, clear water state persisted, even during the Federation drought (1895–1903), between 1891 and 1930, but the episodic floods in 1917 kept the Billabong wet supporting a relatively resilient hydrology^61^. However, climate, and the deliberate pumping of water into the basin allowed gradual sedimentation and the accumulation of biological and chemical remains in the bottom sediments as revealed by both the dated sediment core and preserved cladoceran subfossils along with their carbon and nitrogen isotopes. The relative abundance of littoral taxa such as *D. crassa,* and the increased littoral to planktonic (L:P) ratios of cladocerans during this time (Fig. 3), suggest that both the Murray River and Kings Billabong were dominated by a submerged macrophyte community with high water clarity^33^. The pumping of river water via the Psyche Bend during this period played an important role in maintaining the water level as well as supporting diverse floral and faunal communities, and retaining subfossil cladocerans, and suspended sediments including carbon and nitrogen in Kings Billabong.

#### 1930–1970 AD

After 1930, the influence of anthropogenic disturbance in the Kings Billabong wetland ecosystem increased. A high abundance of *B. meridionalis,* with reduced density of littoral cladocerans, are indicative of increased disturbance with the onset of changes in water quality (Fig. 3)^62^ which coincided with river regulation and land use intensifications across northwest Victoria^63^. Onset of riverbank erosion caused by forest clearances and increased household and animal wastes led to the variability in the flux of sediments including carbon and nitrogen into the wetland. However, efforts were also made in waste management practices from the local government to avoid water pollution further. The rate of ecosystem change in the wetland during this time was relatively slow indicating ecological resistance and the integrity of the source water from the Murray River^33^. Natural ecosystems often show resilience until an abrupt change happens^64^. However, gradual change in the ecosystem condition with dissolved organic carbon (DOC) concentration and a reduced light regime can lead to the loss of submerged macrophytes and diversity in the invertebrate faunas. In Kings Billabong, the cumulative impacts of drivers were not strongly visible at this period. However, the 1930s, the time of the major phase of weir construction for river regulation, was the point at which the system began to change. The water quality, ecosystem and food web of the wetland, all responded to the impact of river regulation. The reduction in the δ^13^C values in *Daphnia* after 1930 (Fig. 4) is an indication of the simplification of the food web that resulted from a gradual switch of *Daphnia* food sources derived from different primary producers.

The dynamics of carbon at the base of the food web in shallow lakes is influenced largely by the submerged macrophyte density and the periphyton attached to it, and phytoplankton. The primary producers utilize available dissolved inorganic carbon (CO_2_ or HCO_3_^−^) with varying isotopic signatures in the wetland pool during photosynthesis^18^. In the presence of submerged macrophytes, periphyton has a wider boundary layer and corresponding reduced CO_2_ uptake than free floating phytoplankton, and as such, have enriched ^13^C isotopes^25^. This is due to increased fixation of HCO_3_^−^ rather than CO_2_^27^ a phenomenon reflected in Kings Billabong prior to the 1930s, when there were abundant submerged macrophytes. However, when the wetland went through a change to lower to moderate primary productivity, algae were able to utilize the lighter ^12^C isotope preferentially from the available CO_2_ pool resulting in the depleted δ^13^C^31^. Most suspended organic matter, composed of phytoplankton, plant detritus, and riverine organic matter, was likely to have been the diet of *Daphnia* in Kings Billabong after the 1970s as indicated by depleted ^13^C^27^. Changes of the dynamics of the riverine organic matter derived from C3 and C4 plants from the catchments can also alter δ^13^C values in sediment over time^65^. Further, in simplified food webs, trophic links between primary producers and primary consumers are reduced^66^, and the ^13^C enriched macrophyte may be replaced by ^13^C depleted phytoplankton under hydrological regulation^67^. As a result, consumers, such as *Daphnia*, are forced to rely on single and poorly resourced phytoplankton food^27,66^. This is also consistent with previous studies that show *Daphnia* to switch their diet when food sources in the system change due to natural and anthropogenic disturbances^17,68,69^.

#### 1970–2011 AD

This period experienced a further gradual decline in ecosystem condition with the further shift to ^13^C carbon depleted-food sources to *Daphnia*. By the 1980s the food web was represented by highly depleted ^13^C in *Daphnia* and by 2011, the Kings Billabong ecosystem had shifted to one driven by low quality diet such as phytoplankton and fringing macrophytes.

### Food web dynamics: causes and consequences

The submerged macrophytes at the base of the food web of the early Kings Billabong ecosystem played an important role in supporting littoral cladocerans^18^ and carbon and nitrogen dynamics across the trophic levels. In the presence of sunlight, the fish, cladocerans and vegetation interactions and external nutrient inputs maintained ecosystem processes. Sun light increases underwater photosynthesis and carbon and nutrient recycling^70^ while *Daphnia* utilizes both littoral and planktonic habitats for food and to avoid predators^32,71,72^. In Kings Billabong, the macrophyte-derived DOC transferred to higher trophic levels via *Daphnia* and other zooplankton in the post 1930s, was different to that in the pre-1930s^25^. During the post 1930s, and particularly after the 1970s, the decline of the submerged littoral vegetation community triggered a shift in the food web structure. Low abundance of *D. crassa,* a littoral cladoceran taxon, indicates a profound disruption in the base of the food web and fish diet after the 1930s. Studies suggest that exotic common carp in the Murray river system intensify submerged macrophyte losses, increase competition for food among native fish community^15^ and alter predator–prey interactions^73^. The reduced δ^13^C values in subfossil *Daphnia*, subfossil chironomid and bulk sediments over time indicate the rapidly changing nature of food sources at the base of the food web (Fig. 4). Relatively higher values of δ^13^C in bulk sediment, compared to that in subfossil *Daphnia* and chironomids (Fig. 4), suggest that the sediment carbon in Kings Billabong is composed of a combination of allochthonous (catchment C3 and C4 plants) and autochthonous organic matter that accumulated in the catchment over a longer period^65^ and is weakly associated with *Daphnia*. Primary producers and other allochthonous organic matter pools in the seston and sediments often confound δ^13^C and δ^15^N measurements of the consumer food source^74^. Climate change also intensifies food webs and top-down and bottom-up processes by affecting consumer physiology and their feeding behavior with effects on resource nutrient contents including the stable isotopes of carbon and nitrogen^69^.

The widespread deforestation of eucalypt trees over the past century, increased erosion and sedimentation, variation in the carbon production^75,76^, interruption in the river-wetland connectivity after 1930 and subsequent resource movements within the wetland, destabilized the food web processes^77^. In the mixing model (Fig. 5), the δ^13^C values of *Daphnia* and zooplanktivorous fish (e.g. Australian smelt) show a very weak relationship indicating a switch of diet by the fish. If Australian smelt were predated by exotic common carp, as revealed by the δ^13^C values of the carp, the consequences in the trophic interactions between zooplanktivorous fish and *Daphnia,* and subsequent change in the food web structure, would not be easily verified. Differential feeding and predator avoidance behavior of *Daphnia,* and various environmental factors including river regulation and climate change, likely led to a switch of food web in Kings Billabong over time by replacing ^13^C enriched submerged macrophytes with ^13^C depleted phytoplankton (Fig. 5). The increased density of emergent macrophytes *T. orientalis*, *T. domingensis* and their δ^13^C values do not show a relationship with *Daphnia* and fish (Fig. 5a) suggesting that the mechanism behind the transfer of carbon from these plants is yet to be established.

The δ^15^N values of primary producers and consumers suggest that modern *Daphnia* may be receiving nitrogen from emergent macrophytes and this contributes to higher trophic levels including those of Australian smelt and Murray cod (Fig. 5a). The increase, over 5 years, to higher mean δ^15^N values in modern *Daphnia,* compared to that in subfossil *Daphnia* indicates a rapid nutrient enrichment shift and changes in the wetland ecosystem and its trophic interactions. Lakes with rapidly changing ecosystems, and increased concentrations of TP, elsewhere also show higher values of δ^15^N^78^. However, the degree of trophic interactions, as a result of nutrient enrichment in Kings Billabong, is not known. For example, the δ^15^N values of emergent macrophytes are the lowest suggesting the source of nitrogen to *Daphnia* could also be from plants other than the emergent macrophytes. The *Daphnia* population also appears to be increasingly vulnerable due to changes in the nutrient status of the wetland in recent times. There is a possibility that the replacement of *Daphnia* by other less vulnerable prey may lead to further predation intensification of common carp resulting in more stabilized trophic levels^79^. Such changes in the condition of the Kings Billabong ecosystem require further investigation.

### Implications for management

A sustained transformation of water quality and ecosystem of Kings Billabong over the past century is indicative of the need of strong measures for wetland management in southeast Australia. Although the response of cladocerans, as revealed by their subfossil records in sediments, is attributed to a localized condition change, the changes are also likely to be associated with catchment level or basin scale modifications of land and water including the introduction of invasive species of common carp causing increased sediments and nutrient fluxes into the Murray River system, sediment resuspension and subsequent reduction in water clarity of the connected wetlands.

Like the proxies used to indicate wetland condition elsewhere^21,80,81^, our records suggest that the Kings Billabong ecosystem was once rich in biological diversity. The degradation of the ecosystem, the reduction in the diversity of its flora and fauna and the simplification of its food web is likely not attributable to proximal causes. Locally managed ecosystem practices may mitigate the drivers of change to a certain degree but the quality of the river itself remains the main cause of the Billabong’s decline. The trophic dynamics between producers and various consumers suggest that the gradual decline in the ecosystem, began in 1930, and owing to the recent changes, appears not to have reached to an endpoint yet. Being once intermittent, but now permanently inundated, the mere provision of water will not lead to the recovery of the diversity this wetland once supported. Management outcomes that may recover the rich community of the past will include a strong focus on recovering stands of submerged plants by improving the quality of the Billabong’s water, it being nourished by the Murray River catchment, by mitigating the nutrient and sediment flux.

The approach used in this study provides evidence of ecosystem processes and food web dynamics that can have implications for the management of wetland ecosystems. The δ^13^C and δ^15^N mixing models derived from modern and subfossil biota reveal much about the contemporary and past dynamics of the regulated river system in southeast Australia. More broadly, the approach also reveals in detail the nature of the switching of trophic positions, of primary and secondary consumers, when exposed to external drivers including nutrients, hydrological modification, sedimentation and invasion.
